# The Diagnostic Potential of Urinary Titin Fragment in Neuromuscular Diseases

**DOI:** 10.3390/ijms26199652

**Published:** 2025-10-03

**Authors:** Andrea Sipos, Dávid Varga, Endre Pál

**Affiliations:** Department of Neurology, University Medical School of Pécs, Rét u. 2, 7623 Pécs, Hungary; sipos.andrea@aok.pte.hu (A.S.); varga.david@pte.hu (D.V.)

**Keywords:** biomarker, cardiomyopathy, myopathy, muscular dystrophy, urinary titin

## Abstract

Biomarkers are important for the diagnosis and follow-up of neuromuscular diseases. Creatine kinase (CK) is a widely used marker of active muscle damage; however, it is not suitable for assessing muscle mass loss. Therefore, additional biomarkers are required to monitor skeletal muscle damage and loss. Titin plays an essential role in the structure and function of muscle fibers. It provides stability and elasticity to the sarcomeres. During sarcomere damage, fragments of titin and other proteins are released from muscle fibers and can be detected in blood and urine. Urinary titin-N fragment (UTN) detection is a noninvasive method for assessing and monitoring the extent of muscle damage. In addition to muscular dystrophies, elevated UTN levels have been observed in patients with sarcopenia. The UTN level increased significantly during eccentric muscle strain, indicating muscle damage, whereas the concentric load was associated with only a minimal increase in UTN. As titin is also present in the heart muscle, UTN can help diagnose cardiomyopathies and predict disease prognosis. In summary, the detection of urinary titin fragments is a promising tool for diagnosing and monitoring neuromuscular and cardiac diseases. While both CK and UTN rise and are related in acute conditions, their relationship is less clear in chronic diseases where muscle tissue damage and muscle mass loss are combined.

## 1. Introduction

### 1.1. Biomarkers

Biomarkers offer various applications in medical research and clinical practice. They provide objective measures of biological processes, disease states and responses to treatment. Therefore, biomarkers may be important in diagnostic procedures and can help predict disease progression and treatment outcomes. Additionally, they often offer noninvasive monitoring options. However, biomarkers have their limitations. However, many of these methods lack sufficient sensitivity or specificity for reliable clinical use. The development and validation of biomarkers is time-consuming and expensive. Furthermore, the complex nature of many diseases means that single biomarkers may not capture the full picture, necessitating the use of multiple markers.

Ideal biomarkers must meet several key requirements to be considered reliable and useful: 1. Specificity: The biomarker should be uniquely associated with a specific biological process or disease. 2. Sensitivity: It must be detectable at relevant concentrations and able to distinguish between normal and abnormal states. 3. Reproducibility: The results should be consistent across different laboratories and testing conditions. 4. Stability: Biomarkers should remain stable during sample collection, storage and analysis. 5. Measurability: It should be quantified using standardized, cost-effective methods. 6. Clinical relevance: Biomarkers must provide meaningful information that impacts patient care and research outcomes. 7. Predictive value: The biomarker should accurately indicate disease risk, progression, or treatment response. 8. Noninvasiveness: The biomarker should be detectable in easily accessible specimens, such as blood, urine, or other bodily fluids, ideally requiring minimally invasive procedures [1].

### 1.2. Biomarkers in Neuromuscular Disorders

#### 1.2.1. Biomarkers of Muscle Mass, Sarcopenia and Injury

Research on skeletal muscle biomarkers has a long history. In the last decade, several investigations have been conducted at various levels.

Muscle mass can be measured using a number of ways, including imaging, anthropometric, electrical methods, as well as chemical assays of blood and urine and finally by assessing physical performance [2].

Body mass index (BMI), skinfold thickness, and waist–hip ratio are simple methods, but they are more closely related to body fat than to muscle; therefore, they do not provide direct information on muscle mass. While dual-energy X-ray absorptiometry (DEXA) requires special equipment and X-rays, bioelectrical impedance analysis (BIA) is an easy and inexpensive method for estimating skeletal muscle mass; the latter is influenced by many factors, and these methods do not reflect disease activity [3].

Among the biochemical parameters, serum creatinine concentration was found to be associated with muscle mass. The majority of creatinine is produced by the skeletal muscles and excreted exclusively in the urine. In clinical practice, serum creatinine level is commonly measured, but it depends on kidney health; therefore, it is a suitable marker only in subjects with intact kidney function. Another possibility to determine the creatinine/cystatin C ratio is an alternative filtration marker (recent review by Liu et al. [4]).

Quantitative magnetic resonance imaging (qMRI) has been implemented for skeletal muscle investigations using various techniques. This requires manual segmentation or a recently introduced semi-automated mode supported by artificial intelligence. In clinical settings, T1-weighted and Dixon sequences are commonly used for segmentation and identification of individual muscles and separation of internal, extramuscular, and subcutaneous fat fractions [5]. However, this method does not reflect functional changes.

#### 1.2.2. Biomarkers of Acute Muscle Damage

Acute skeletal muscle damage can occur under a variety of conditions. Typically, eccentric exercises cause microdamage to muscle fibers. Muscle damage develops under ischemic conditions due to toxic agents and extreme physical loads. Rhabdomyolysis is the most severe form of skeletal muscle damage.

The assessment of acute muscle injury is challenging. Histological investigations (biopsy) are inconvenient and may not represent the entire musculature. MRI is sensitive, but the alterations are non-specific and may indicate either edema or micro-injury. Delayed-onset muscle soreness (DOMS) is a common consequence of excessive load; however, its severity is subjective and depends on individual factors. Therefore, these markers are not suitable for routine monitoring of muscle injury and recovery [6].

The most commonly used blood-derived marker is the serum creatine kinase (CK) level. In acute skeletal muscle injury, CK peaks 24–72 h after the onset of damage and gradually decreases, finally normalizes 5–10 days later, depending on the maximum value [7]. Although CK reflects disease activity, it does not provide information on muscle mass. Furthermore, in patients with severe muscle atrophy, CK levels tend to be normal (personal observation).

Theoretically, a number of other soluble markers can be found in the blood and urine released from the damaged muscle, including sarcomeric proteins, such as troponin I, myosin, myomesin, and myoglobin, if the lesion is severe, as seen in rhabdomyolysis. The determination of the above-mentioned markers is more difficult than that of CK, which is easily accessible in all laboratories [8].

### 1.3. Titin Structure and Function

Titin, the largest known protein in vertebrate muscles, plays a central role in maintaining sarcomere elasticity. Over the past few decades, research has elucidated multiple facets of titin, from its tissue-specific splicing to its role in assembling and maintaining the sarcomere and its regulated degradation.

Titin is encoded by the *TTN* gene, one of the longest genes in the human genome, which comprises more than 360 exons. Research has revealed that titin transcript encodes a protein that may exceed 30,000 amino acids in length having a molecular weight ranging from approximately 3–3.8 MegaDaltons (MDa), and the full length of titin, depending on the isoform, ranges from 1.0 to 1.2 μm. This size allows titin to span half of the sarcomere from the Z-disk at one end to the M-line at the other end, providing structural integrity and elasticity. Alternative splicing of TTN exons generates several isoforms that vary in length and elasticity because of the differential inclusion of I-band exons, which encode modular elements such as immunoglobulin (Ig)-like domains and proline, glutamic acid, valine, and lysine-rich (PEVK) segments. The development of these isoforms is tissue-specific. Fetal muscles express longer and more compliant titin variants, whereas adult muscles, especially those of fast-twitch fibers, express shorter variants. Titin isoforms play a crucial role in determining the mechanical properties of muscle tissue [9].

In cardiac muscle, the two main isoforms, N2B and N2BA, differ significantly in their structures and functions. The N2B isoform is shorter (3.0 MDa) and stiffer, contributing to the higher passive tension observed in adult cardiac muscle, which is essential for efficient contraction and relaxation cycles. In contrast, the N2BA isoform is longer (3.3–3.5 MDa) and more compliant, allowing for greater elasticity and stretchability within the myocardium. The ratio of N2B to N2BA isoforms is 60:40 in adult myocardium, but it differs in species [10]. It can shift in response to developmental cues (during the perinatal period), physiological demands, or pathological conditions such as heart failure or cardiomyopathy, where an increased expression of the more compliant N2BA isoform is often observed. This shift can serve as an adaptive mechanism to cope with increased cardiac workload or altered hemodynamics [11,12].

In skeletal muscle, the predominant isoform is N2A (with a molecular weight approaching 3.7–3.8 MDa), which provides a balance between elasticity and stiffness suitable for the rapid and forceful contractions required in these tissues [9,10].

The study of titin isoforms has provided valuable insights into muscle physiology and disease, highlighting their importance in health and pathology. The dynamic regulation of titin isoform expression and modification underscores the central role of this protein in muscle adaptability and resilience.

### 1.4. Sarcomere Structure and Titin’s Role

**Figure 1:**
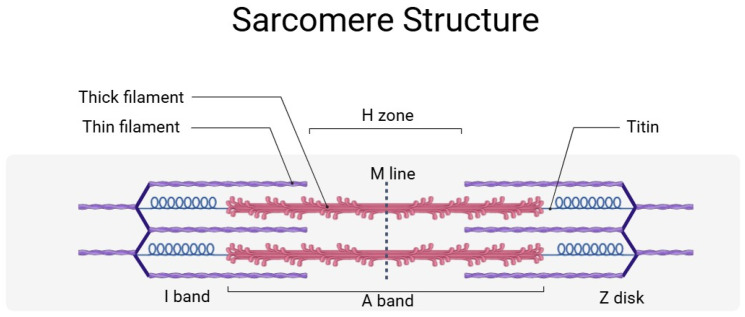
Simplified sarcomere structure with titin. Titin anchors the sarcomere from the Z-disk to the M line and participates in the maintenance of the sarcomere structure and elasticity. A detailed description is provided in the text. This figure was created by BioRender (app.biorender.com, accessed on 3 June 2025).

The sarcomere, the basic contractile unit of muscle, is organized into a highly ordered array of thick and thin filaments interlinked by titin. Thick and thin filaments are composed mainly of myosin and actin, respectively (Figure 1) and are associated with other proteins. In the Z-disk, the N-terminal region of titin contains repeated motifs known as Z-repeats, which bind to proteins such as α-actinin. This interaction anchors titin to the Z-disk and contributes to the lateral stability of the sarcomere by crosslinking actin filaments. The I-band region of titin comprises a series of Ig-like domains interspersed with an extensible PEVK segment. This region is primarily responsible for the passive elasticity of the muscle, and, as the muscle stretches, the I-band domains gradually unfold and extend, thereby generating a restoring force that helps return the sarcomere to its resting length. The precise number and arrangement of these domains are modulated by alternative splicing, which is essential for tailoring the passive stiffness of different muscles. Within the A-band, titin is more rigid. This region contains multiple super-repeats of Ig and fibronectin type—3 (FN3) domains. Here, titin might function as a molecular ruler, ensuring that the thick filament maintains a constant length and that the spacing of myosin heads is optimized for efficient cross-bridge formation during contraction. At the M-line, the C-terminal end of titin interacts with myomesin and other M-line proteins to secure thick filaments in place. This modular organization not only provides mechanical stability but also enables titin to function as a scaffold for signaling molecules that monitor mechanical stress and coordinate muscle remodeling [9,10].

Beyond titin–isoform switch, posttranslational modification (PTM) is responsible for further modification. The extensible regions of titin are targets for phosphorylation; in addition, calcium binding has been shown to modulate the conformation of certain titin domains. Recent research uncovered further evidence of PTM including acetylation/deacetylation, and oxidation/reduction, involving not only titin but other cardiac muscle proteins as well (detailed review is found in [13]).

Titin plays a critical role in maintaining sarcomeric stability and protein turnover. In adult mice, the half-life is 2–3 weeks. On one side, chaperones (mainly heat shock proteins) stabilize titin to prevent stiffening; on the other hand, if chaperones fail to protect the protein, it becomes marked for degradation and turnover. The titin kinase (TK) domain is an important locus for the cross-talk of sarcomere stretch signals and cellular turn-over factors, namely the autophagosomal receptors Nbr1, p62 and MuRF E3 ubiquitin ligases. MuRF2/Nbr1/p62 complex formation onto TK was proposed to occur in active muscle [14]. In contrast, an alternative TK-based assembly consisting of MuRF1/Nbr1/p62 occurs in the absence of stretch and is dependent on MuRF1-mediated ubiquitination of TK. Thus, ubiquitination increases in inactive muscle, and autophagy process is mediated by Nbr1/p62. This process leads to the breakdown of sarcomeric proteins, including titin, finally causing sarcomeric instability, protein loss and muscle atrophy [14,15].

### 1.5. Development of Urinary Titin-Fragment Diagnostics

#### 1.5.1. Discovery of Urinary Titin Fragments

The identification of titin fragments in urine was a significant breakthrough in biomarker research on muscular disorders. This discovery was initiated by proteomic profiling of urine samples from patients with Duchenne muscular dystrophy (DMD) and healthy individuals. Comparative analysis using liquid chromatography-tandem mass spectrometry revealed that titin was one of the most significantly elevated proteins in the urine of DMD patients. Western blot analysis confirmed the presence of N- and C-terminal titin fragments in urine samples from patients with DMD but not in those with neurogenic disorders, such as spinal muscular atrophy. This suggests that titin fragments can serve as non-invasive biomarkers for monitoring muscle degradation [16].

#### 1.5.2. Development of ELISA for Urinary Titin Determination

Given the potential of titin as a biomarker, Maruyama’s group developed a sensitive enzyme-linked immunosorbent assay (ELISA) to quantify the N-terminal titin fragment in urine (UTN) [17]. The assay was validated against mass spectrometry and Western blot techniques, confirming its specificity and reliability. One of the key advantages of this ELISA system is its ability to detect titin fragments at extremely low concentrations, making it a valuable tool for screening and monitoring muscle diseases. The sensitivity and specificity of the ELISA were outstanding, with an area under the curve (AUC) value of 0.9992 in distinguishing between DMD patients and controls. The ELISA system was applied to measure urinary titin concentrations in healthy individuals to establish reference values. The mean urinary titin-N fragment concentration in healthy adults ranges from 1.1 to 7.9 pmol/mg creatinine (Cr). The authors determined the inter-assay variations as well as circadian fluctuations, both of which were minimal, indicating that a single urine sample could provide reliable diagnostic information [17].

## 2. Overview Urinary Titin-N-Fragment Diagnostics in Neuromuscular Diseases

Neuromuscular diseases encompass a broad spectrum of conditions that affect muscle structure and function. These disorders typically result in muscle atrophy, weakness, fatigue, and reduced mobility, significantly compromising the patients’ quality of life. The following sections highlight the findings related to the role of UTN in improving the diagnosis and treatment approaches for key neuromuscular disorders.

### 2.1. Urinary Titin-N Fragment in Muscular Dystrophies

Titin degradation products have emerged as potential biomarkers of muscle damage in various muscular dystrophies (MDs). These genetic disorders are characterized by progressive muscle degeneration, atrophy, and muscle weakness. Titin fragments have been detected in the urine of patients with various forms of muscular dystrophy, indicating their potential role in disease assessment and monitoring.

The release of titin into urine is attributed to muscle fiber breakdown through several mechanisms: (1) dystrophin deficiency: in conditions such as DMD, sarcolemma instability leads to increased muscle damage and leakage of intracellular proteins, including titin; (2) calcium dysregulation: dystrophic muscle fibers experience excessive calcium influx, activating proteases that cleave titin into fragments that are subsequently excreted in urine; and (3) exercise and mechanical stress: studies have shown that eccentric exercise (which lengthens muscle fibers under load) induces myofiber damage and urinary titin release [6].

### 2.2. Duchenne Muscular Dystrophy

DMD is the most common and severe childhood-onset muscular dystrophy, caused by mutations in the *DMD* gene, resulting in dystrophin deficiency, a crucial protein for muscle fiber stability. The disease primarily affects boys, with symptoms manifesting in early childhood. Progressive muscle weakness leads to loss of ambulation by the early teens and eventual death due to respiratory and cardiac insufficiency around the age of 20 to 30 years.

Studies have demonstrated significantly elevated UTN levels in the urine of patients with DMD. Robertson et al., 2017 showed urinary titin levels 365 times higher in DMD patients compared to healthy individuals, while Matsuo et al., 2019 found increases of up to 700-fold [18,19]. This elevation is attributed to the excessive muscle degradation observed in DMD due to dystrophin deficiency. UTN levels correlated with other markers of muscle damage, such as serum creatine kinase, and tended to decrease with age, making UTN suitable for tracking disease progression. Additionally, ambulatory patients had higher rates than non-ambulatory patients. AUC was calculated to distinguish DMD from controls, and it was 1.0 with a cut-off value of 3.84 pmol/mgCr [18,19].

Comparable results were found in an animal model of DMD: muscular dystrophy X-linked (mdx) mice. UTN levels were determined using a mouse titin-specific ELISA kit. Five-hundred-fold higher UTN levels were measured in mdx mice than in healthy mice. This study found an age-related decrease in UTN, similar to the results of human studies [20].

### 2.3. Becker Muscular Dystrophy (BMD)

BMD represents a milder variant of dystrophinopathy, also caused by *DMD* gene mutations, but with partially functional dystrophin expression. Symptoms typically appear in adolescence, with slower disease progression than DMD. Many patients maintain ambulation into adulthood, although cardiac issues remain a significant concern. Although lower than that in DMD, urinary titin levels in patients with BMD still exceed 120 times that of healthy subjects. The AUC for distinguishing BMD from DMD was 0.8814, with a cutoff of 619.5 pmol/mgCr [20,21,22].

The results suggest that UTN correlates with muscular dystrophy activity, specifically with the speed of muscle breakdown. These studies showed that UTN levels are variable, being extremely high in the early active disease stage, while they might be normal in advanced, inactive disease. Furthermore, UTN helps differentiate DMD from BMD.

### 2.4. Limb-Girdle Muscular Dystrophy (LGMD)

LGMD encompasses a diverse group of disorders affecting the pelvic and shoulder girdle muscles, resulting from mutations in various genes. These conditions exhibit both autosomal dominant and recessive inheritance patterns. Disease severity varies considerably, with some individuals experiencing only mild weakness, whereas others lose their mobility earlier in life. In a pioneering study by Rouillon et al., elevated UTN levels were found in nine patients with different LGMD types [16]. Unfortunately, there were no further studies to confirm the results found in this small and heterogeneous patient population.

### 2.5. Fukuyama Congenital Muscular Dystrophy (FCMD)

FCMD is a rare autosomal recessive disorder prevalent in Japan caused by mutations in the FKTN gene. This condition is characterized by severe muscle weakness from birth, brain abnormalities, and developmental delay. Most patients experience significant motor impairments and reduced lifespan due to respiratory complications. Urinary titin levels in FCMD patients have been found to be more than 320 times higher than control values from previous studies. UTN was moderately correlated with serum CK levels and clinical scores [23].

### 2.6. Myotonic Dystrophy Type 1 (DM1)

Myotonic dystrophy is a multisystem disorder caused by mutations in the DMPK (DM1) or CNBP (DM2) genes, leading to progressive muscle wasting, cardiac issues, and other systemic complications, such as cataract and insulin resistance. It is the most common adult-onset muscular dystrophy, with varying severities of genetic alterations. A recent study demonstrated that DM1 patients exhibit significantly elevated, more than five times higher, UTN levels compared to healthy individuals. In this study the sensitivity and specificity of the measurement at 12.267 pmol/mgCr cut-off value were 0.966 and 0.967, respectively, with AUC = 0.989. Although CK levels were elevated, only the disease-specific Muscle Impairment Rating Scale (MIRS) correlated moderately with UTN [24]. This study investigated a heterogeneous adult patient population with advanced clinical disease stages. This might explain the smaller difference in UTN levels between patients and controls (in contrast, DMD and FCMD studies investigated younger patients).

Table 1 summarizes the UTN studies on muscular dystrophies.

Urinary titin is a highly promising biomarker for monitoring muscular dystrophies, such as DMD, BMD, FCMD, and DM1. The development of an ELISA-based urinary titin test represents a major advancement in the noninvasive screening and follow-up of muscular dystrophies. Considerable differences in UTN have been observed in different muscular dystrophies, reflecting variations in muscle damage and disease activity. There are limitations to muscular dystrophy studies. As mentioned, there were considerable age differences (adults in DM1 versus children in DMD and FCMD). Heterogeneous patient populations were included (walkers and non-walkers in DMD), and all studies were cross-sectional. Future research will likely focus on refining these assays and validating urinary titin in clinical settings, particularly in LGMDs, where only a few cases have been documented, and in other rare forms of muscular dystrophy. Long-term follow-up studies are required to determine the usability of UTN in the progression of muscular dystrophies.

### 2.7. Urinary Titin-N Fragment in Sarcopenia

A study involving 39 patients with gastrointestinal tract and hepatobiliary–pancreatic cancers found elevated UTN levels in patients with sarcopenia compared to those without sarcopenia (8.3 and 4.9 pmol/mgCr, respectively). UTN is correlated with preoperative sarcopenia and poor nutritional status. The study demonstrated significant negative correlations between titin concentrations and key nutritional and muscle mass indicators, including albumin, prealbumin, BMI, and skeletal muscle volume index. The authors determined that urinary titin may serve as a practical biomarker for evaluating both sarcopenia and overall nutritional health [25].

In another study focusing on individuals with type-2 diabetes mellitus (T2DM), UTN levels were significantly higher in patients with diabetes than in non-diabetic controls, even after matching for age, sex, and BMI (odds ratio, 2.46). Notably, in elderly men (≥75 years), high titin levels were strongly associated with sarcopenia (odds ratio, 6.61). The results indicated that T2DM itself was associated with a “high-titin state,” reinforcing the concept that diabetes may accelerate muscle degradation, thereby increasing the risk of sarcopenia [26].

One study assessed 68 patients with interstitial lung disease (ILD) to determine whether UTN levels could predict longitudinal muscle loss in these patients. In this cohort, the mean UTN was 7.0 pm/mgCr. Higher titin levels were significantly and negatively correlated with changes in muscle cross-sectional areas measured from CT scans of the pectoral and erector spinae muscles over 6 months to 1 year. These findings support the use of urinary titin as a prognostic biomarker to identify patients with ILD at risk of progressive muscle wasting, which is known to contribute to poor clinical outcomes [27].

Ishihara et al., 2021 investigated patients after stroke [28]. They reported that the level of urinary titin N-fragment was correlated with functional impairment. Peak UTN levels of 41 patients during the seven days of admission were correlated with the modified Rankin scale score (r = 0.55, *p* < 0.01), National Institute of Health stroke scale value (r = 0.72, *p* < 0.01), and Barthel index (r = − 0.59, *p* < 0.01) at the time of hospital discharge. In the multivariate analysis adjusted for the disease severity, the UTN on day 2 predicted the functional outcome at hospital discharge (odds ratio, 1.11; 95% CI, 1.01–1.28). UTN levels reflect muscle breakdown and subsequent functional impairment [28].

Collectively, these studies demonstrate that urinary titin is a promising noninvasive biomarker for the early detection of muscle loss across a spectrum of conditions. Titin levels reflect the extent of muscle damage and patient’s nutritional status. Future research should refine titin threshold values specific to sarcopenia because UTN levels in cancer and ILD patients were in the normal range, but stroke patients had UTN levels above the accepted 12 pmol/mgCr threshold. Further research is needed to determine how different diseases influence titin breakdown. The results will hopefully evaluate the role of UTN in guiding therapeutic interventions aimed at preserving muscle mass.

### 2.8. Urinary Titin-N Fragment in Acute Conditions

#### 2.8.1. Intensive Care Unit-Acquired Weakness (ICU-AW)

ICU-acquired weakness includes critically ill patients exposed to prolonged mechanical ventilation, immobilization, or systemic inflammation, resulting in muscle atrophy and weakness.

A previous study investigated UTN levels on days 1, 3, 5, and 7 after admission to the ICU. The mean UTN in 50 patients was 50.60 pmol/mgCr (range 23.97–111.69), which was significantly higher than the normal levels found in healthy subjects from earlier studies. A high UTN was associated with weakness, indicating acquired critical illness myopathy. These results suggest that UTN may help assess the presence and progression of critical illness [29].

Nakanishi et al., 2020 investigated UTN in 56 critically ill patients and found that the cumulative UTN up to discharge or day 7 was higher in ICU-AW than in non-ICU-AW patients [314.1 (range 181.5–464.7) vs. 86.6 (range 66.3–171.1 pmol/mgCr), *p* = 0.01] [30]. Their study demonstrated that urinary titin levels on day 2 predicted ICU-AW with a sensitivity of 78% and specificity of 81% at a cut-off value of 64.8 pmol/mgCr [30].

#### 2.8.2. Urinary Titin-N Fragment in Acute Skeletal Muscle Injury

UTN was determined in samples from 62 patients with idiopathic inflammatory myopathy (IIM), 59 patients with other connective tissue disorders (CTD), and 29 healthy controls. The UTN level of the IIM group [168.3 (range 19.0–1279.0) pmol/mgCr] was significantly higher than that in the CTD controls [2.80 (range 1.53–3.60)] and healthy controls [1.83 (range 1.09–2.95) pmol/mgCr] (*p* < 0.001). IIM patients with skeletal muscle injury had a significantly higher level of urinary N-titin [1001.0, (181.8, 1977.0)] than those without [9.3, (5.8, 23.9) pmol/mgCr] (*p* < 0.001) (all data are expressed as median (IQR)). UTN level was strongly correlated with CK (r = 0.907, *p* < 0.001), suggesting that the urinary N-titin fragment is a noninvasive and independent predictive factor for determining skeletal muscle injury in patients with IIM [31].

A single-center study evaluated the UTN in patients with mild injuries. The values of N-titin/Cr of untreated patients were 20.6 and 18.2 pmol/mgCr, on days 1 and 3, respectively. This study did not use healthy control data, but it did use the same ELISA method, suggesting that these values were elevated [32].

#### 2.8.3. Urinary Titin After Exercise

Urinary titin fragments have also been identified as non-invasive biomarkers of exercise-induced muscle damage. Multiple studies have highlighted the relationship between urinary titin levels and various types of exercise. Urinary titin fragments appear in response to muscle damage caused by eccentric exercises. The increase in urinary titin levels is strongly correlated with blood markers such as CK and myoglobin. Studies have shown that the urinary titin concentration increases similarly to that of CK but may appear slightly earlier. This earlier appearance may be due to the smaller molecular weight of titin fragments compared to CK, allowing faster excretion through urine [6].

#### 2.8.4. Exercise Type and Urinary Titin Response

Distinct types of eccentric exercise, such as drop jumps and eccentric ergometer training, increase urinary titin levels. However, concentric exercises do not lead to similar increases, indicating that urinary titin levels reflect muscle damage, specifically from eccentric contractions. UTN was measured in nine individuals before and after eccentric exercise and was found to increase to 96 h after the exercise (5.1 to 77.6 pmol/mgCr, *p* < 0.01). The levels of urinary titin fragment were significantly correlated with muscle damage symptoms, including DOMS, reduced muscle strength, range of motion (ROM), and changes in serum CK and myoglobin [33].

To investigate exercise-induced muscle damage, UTN was determined in 9 healthy subjects after concentric and eccentric exercise. UTN was measured immediately after exercise and at 24, 48, 72, 96, 120, and 144 h after exercise. The peak percentage of UTN changes occurred at 96 h after exercise. UTN was significantly higher after eccentric versus concentric exercise in all measurement points; eccentric (9.67–449.67 pmol/mg/dl) versus concentric (1.49–3.58 pmol/mg/dL) (*p* < 0.001) [34].

Lee et al., 2021 investigated healthy young male subjects who performed drop jump (*n* = 9) and eccentric ergometer exercise (*n* = 9) [35]. Blood and urine samples were collected 24 and 48 h after both interventions. Levels of the urinary titin fragment, plasma myomesin 3 fragments, CK, and myoglobin were increased after eccentric exercises, but these changes were not statistically significant [35].

Tanabe et al., 2022 investigated UTN before and after isokinetic eccentric contractions of the elbow flexors [36]. UTN gradually increased from baseline to the end of the study period (4 days). UTN concentrations were 16 ± 8 and 645 ± 759 pmol/mg/dl before and 4 days after exercise, respectively (*p* < 0.013) [36].

Investigating an eccentric back squat protocol in 39 individuals, the authors found significantly elevated UTN at 24- and 72-h post-exercise (135.4 ± 32.1% and 104.0 ± 32.1%, respectively) [37].

In a 3000-m running time trial, no significant increase in urinary titin was observed (UTN median (25% quartile, 75% quartile): 5.04 (2.87, 6.49) and 5.27 (2.59, 8.16) ng/mgCr pre- and post-trial, respectively. This study suggests that titin degradation and excretion are not altered during endurance running, whereas the opposite was proven following eccentric exercise [38].

In summary, urinary titin fragments are reliable and noninvasive biomarkers of exercise-induced muscle damage, particularly following eccentric exercise. They correlate well with traditional serum markers, such as CK, and muscle function parameters, making them useful for monitoring recovery in athletes and individuals undergoing resistance training or muscle injuries. The UTN changes depend on the type of injury/exercise and pre—injury conditions (trainees). These results should be interpreted with caution because of the small sample size in all but one [34] of the studies and the different types of exercises.

A summary of these studies is presented in Table 2.

#### 2.8.5. Urinary Titin in Amyotrophic Lateral Sclerosis (ALS)

Compared with 43 healthy controls, significantly increased UTN levels were found in 70 patients with ALS (27.2 pmol/mg/dL versus 5.8 pmol/mg/dL in healthy controls; *p* < 0.001), which showed a moderate correlation with the scores of the Revised Amyotrophic Lateral Sclerosis Functional Rating Scale (*r* = − 0.422, *p* < 0.001). This study demonstrated that high UTN levels served as a survival predictor in patients with ALS. Additionally, motor neuropathy (*n* = 19) and spinal-bulbar muscular atrophy (SBMA, *n* = 69) were analyzed as disease controls. Increased UTN levels were found to be 15.1 ± 13.9 and 190.7 ± 100.5 pmol/mg/dL, respectively. These findings indicate that sarcomere damage results in titin degradation, regardless of whether the cause is primary (muscle) or secondary (denervation). Elevated serum CK levels are a well-known feature of ALS. Interestingly, this study found only a minimal increase in CK, which was not closely related to UTN [39].

Unexpectedly, in the animal model of denervation, UTN did not differ from that in the control group. This study raises the possibility that UTN is a suitable marker of muscle damage in chronic conditions with advanced and/or ongoing denervation but not in acute cases [40].

### 2.9. Urinary Titin-N Fragment in Cardiomyopathy

Mutations in the *TTN* gene have been increasingly recognized as major contributors to various cardiomyopathies, including dilated (DCM) and hypertrophic cardiomyopathy (HCM). Additionally, titin has been implicated in muscular dystrophies that exhibit cardiac complications, highlighting its essential role in cardiac muscle.

In an investigation of various diseases with cardiomyopathy, UTN levels were found to be significantly higher in cardiomyopathy associated with muscular dystrophy than in other types of cardiomyopathy, including sarcoidosis, amyloidosis, and Fabry disease (Table 3) [41].

Recent research has demonstrated the potential of using UTN as biomarkers for diagnosing muscular dystrophy and predicting outcomes in cardiomyopathies. Yoshihisa et al., 2018 measured the urinary Titin-N/creatinine ratios in 102 patients with DCM and followed them for an average of 1167 days [42]. The patients were divided into groups according to their UTN levels. Two-thirds of the patients had normal UTN levels (<7.26 pmol/mgCr), whereas the remaining patients had elevated levels. They found that both cardiac and all-cause mortality progressively increased with increasing UTN. They concluded that UTN serves as a predictor of cardiac and all-cause mortality in patients with DCM, demonstrating that elevated UTN can identify high-risk patients with DCM [42,43].

Main Findings of the UTN studies are summarized in Table 4. These results support the high additional value of the urinary titin test in clinical settings. Different research groups have consistently found that, in normal subjects, UTN is ≤12 pmol/mgCr. Clinical associations were variable, and correlations with serum CK levels were not always proven to be significant. While the sensitivity is between 61.9–100%, the specificity is higher, between 81–100% of the commercially available ELISA test.

### 2.10. Comparison of Serum CK and UTN Levels in Biomarker Studies

Many studies have used CK as a comparator for UNT studies. A brief collection of the results is presented in Table 5. In summary, we conclude that a close relationship and parallel rise in the two markers are demonstrated in acute conditions. In contrast, in chronic conditions, the correlations between CK and UTN are less strong, and, in some studies, absent.

Relationship between serum creatine kinase (CK) and urinary titin fragment (UTN) concentrations is shown under different conditions. Strong correlations were found in acute conditions, whereas, in chronic diseases, the correlation was moderate or absent.

## 3. Discussion

### 3.1. Advantages of Urinary Titin Fragments

Urinary titin fragments have emerged as potential biomarker in clinical practice, particularly for the diagnosis and monitoring of muscle-related disorders. The key applications are as follows.

- Supporting the diagnosis of Neuromuscular Disorders

Elevated urinary titin levels are associated with muscle degradation, making them a valuable marker for conditions such as muscular dystrophies and myopathies [21,24].

- Monitoring Disease Progression

Urinary titin testing captures real-time disease fluctuations that help track the progression of neuromuscular diseases and assess the effectiveness of treatments using a noninvasive approach [37,39].

- Critical illness and Intensive care monitoring

Patients in intensive care units, particularly those with prolonged immobilization or sepsis, often experience muscle atrophy. Urinary titin levels can indicate muscle breakdown and guide rehabilitation strategies [29,30].

- Sports Medicine and Overtraining Syndrome

Urinary titin has been studied as a marker of exercise-induced muscle damage, helping to differentiate between normal training adaptations and pathological muscle injury [35,36,37,38].

- Cardiac Disorders

As titin is a crucial component of both skeletal and cardiac muscles, its presence in urine may be relevant for detecting certain heart conditions, including cardiomyopathies [41,42,43].

### 3.2. Challenges and Opportunities

Cost-effectiveness: As an alternative diagnostic tool, urinary titin testing can complement other approaches, such as blood-derived biomarkers, imaging findings, and electrophysiological investigations.

Standardization: A commercially available, sensitive, and highly specific ELISA method ensures comparability of the results across different studies.

Definition of normal values: The measurement of UTN in healthy controls in different studies has developed a clear-cut normal range. Additional studies are required to demonstrate age-related changes in UTN levels in healthy conditions.

Potential of UTN determination: Disease monitoring and treatment efficacy assessment. Its prognostic value has only been proven in cardiomyopathy and needs to be determined in skeletal muscle diseases.

## 4. Conclusions

Urinary titin fragment analysis is a simple, noninvasive method for diagnosing and monitoring neuromuscular diseases. Its ability to non-invasively track disorders in both chronic (e.g., muscular dystrophies, sarcopenia, and ALS) and acute conditions (such as muscle injury and rhabdomyolysis) offers significant benefits to patients. Implementation of this simple measurement methodology in future clinical trials will firmly establish its value as a biomarker. Studies have shown that both classic biomarkers, such as muscle mass, muscle strength, and serum CK levels, and new measures, such as UTN, have a comparative role in the follow-up of muscle diseases. Assessing the prognostic value of UTN in muscular dystrophies requires serial measurements in follow-up studies.

## Figures and Tables

**Table 1:** UTN studies on muscular dystrophies.

Author	Year	Disease	UTN_p_	UTN_HC_	AUC
Robertson et al. [18]	2017	DMD (*n* = 7)	365.4 ± 65.0 *	1.0 ± 0.4 ng/mL * (*n* = 7)	n.a.
Matsuo et al. [19]	2018	DMD (*n* = 4)	1240.5 **	1.8 ** (*n* = 100)	1.0
Awano et al. [21]	2018	DMD (*n* = 113)	965.8 ± 1011.9 *** [735.5] **	1.4 ± 0.8 *** (*n* = 9)	1.0
		BMD (*n* = 36)	171.2 ± 272.7 *** [43.2] **		0.881
Awano et al. [22]	2025	BMD (*n* = 123)	72.6 **	Compared to previous data (control ≤ 12)	n.a.
Sato et al. [23]	2021	FCMD (*n* = 18)	455.5 ± 243.9 ***	Compared to previous data	n.a.
Varga et al. [24]	2023	DM1 (*n* = 29)	39.3 ± 26.5 ^#^	6.7 ± 5.2 ^#^ (*n* = 30)	0.989

The published studies used different interpretations of the results, but UTN was significantly elevated in all investigations compared to healthy controls. It is widely accepted that UTN in healthy subjects is below 12 pmol/mgCr. The range of UTN concentration is wide among dystrophy patients, implying that certain (advanced) cases have normal values. Data represent **: median, ***: mean ± SD, ^#^: mean ± MAD (Mean Absolute Deviation) in pmol/mgCr; except *: in this study, UTN was not normalized to urinary creatinine. n.a.: not available. The number (*n*) of subjects is in parentheses. AUC: Area under the curve; UTN_p_ and UTN_HC_: Urinary titin fragment concentrations in patients and healthy controls, respectively.

**Table 2:** Investigations of Urinary titin-N fragment (UTN) in distinct types of exercise.

Authors	Year	Type of Exercise	(*n*)	UTN (pmol/mg of Creatinine)
Kanda et al. [33]	2016	Eccentric	9	5.1 (pre); 77.6 (post—exercise, 96 h)
Yamaguchi et al. [34]	2020	Eccentric, concentric	9	Increased after eccentric, but not after concentric exercise
Lee et al. [35]	2020	Eccentric	18	Increased (non-significant)
Tanabe et al. [36]	2021	Eccentric	28	2.1 (pre); 215.4 (post—exercise, 96 h)
Tominaga et al. [38]	2021	Endurance	10	No significant change

*n*: number of patients investigated.

**Table 3:** UTN in cardiomyopathy.

Author		(*n*)	UTN (pmol/mg Cr)
Yoshihisa et al. [41]	DCM	199	4.3 (2.4–8.9)
	HCM	86	3.4 (2.1–6.1)
	Sarcoidosis	18	3.1 (1.9–4.5)
	Amyloidosis	15	5.5 (3.1–11.7)
	Fabry Disease	6	3.9 (2.0–8.6)
	Muscular Dystrophy	7	21.5 (9.0–28.9)

DCM, dilated cardiomyopathy; HCM, hypertrophic cardiomyopathy; *n*, number of patients.

**Table 4:** Summary of the UTN ELISA studies.

Disease	Cut-Off	AUC	Sensitivity	Specificity	Clinical Corr.	Ref.
DMD	3.84	1.0	100	100	CK, age	Matsuo et al. [19]
DMD/BMD	3.52	0.99	98.9	100	DMD/BMD, age	Awano et al. [21]
BMD	12.0	n.p.	n.p.	n.p.	ambulation, TnI	Awano et al. [22]
Fukuyama	n.p.	n.p.	n.p.	n.p.	CK, GMFM	Sato et al. [23]
DM1	12.2	0.989	96.6	96.7	MIRS	Varga et al. [24]
MD + CMP	8.7	0.92	100	82	mortality	Yoshihisa et al. [41]
Sarcopenia	5.2/10.4	n.p.	n.p.	n.p.	PM_CSA_/ESM_CSA_	Hanada et al. [27]
ICU-AW	100	0.81	61.9	89.7	MRC < 48	Nakano et al. [29]
ICU-AW	64.8	0.78	78	81	RF_CSA_	Nakanishi et al. [30]
Myositis	89.9	0.97	87.8	100	CK	Sun et al. [31]

These data indicate that UTN measurements can differentiate between pathological and healthy conditions. UTN cut-off values are in pmol/mgCr (Abbreviations: AUC, area under the curve; n.p., not published; CMP, cardiomyopathy; MIRS, Muscle Impairment Rating Scale; MRC, Muscle Research Council; TnI, serum troponin I; PM/ESM/RF_CSA_, cross-sectional area of pectoral/erector spinae/rectus femoris muscles).

**Table 5:** Comparison of serum CK and UTN as biomarkers.

Condition/Disease	Correlation Strength (CK vs. UTN)	Main Findings	Reference
Exercise-Induced Muscle Damage	Strong, r = 0.868–0.973	Strong positive correlation observed 24–144 h. post eccentric exercise; both markers rise	Yamaguchi et al. [34]
Eccentric exercise	Strong, r = 0.81–0.98	48–75 h. post-exercise (calf-raise)	Kanda et al. [33]
Eccentric exercise	Strong, r = 0.82	On day 1, post-exercise (elbow isokinetic exercise)	Tanabe et al. [36]
Idiopathic Inflammatory Myopathies	Strong, r = 0.907	Both biomarkers rise	Sun et al. [31]
Duchenne Muscular Dystrophy	Strong, r = 0.497	Both biomarkers rise	Awano et al. [21]
Becker muscular dystrophy	Not reported	UTN is a more accurate marker for muscle damage than CK	Awano et al. [22]
Myotonic Dystrophy type 1	No (r not reported)		Varga et al. [24]
Amyotrophic lateral sclerosis	Yes, (r not reported)	log CK/log UTN	Yamada et al. [39]
Sarcopenia	No (r = 0.02)		Miyoshi et al. [25]

## Data Availability

No new data were created.

## Abbreviations

The following abbreviations are used in this manuscript:

AUC	Area under curve
BMD	Becker muscular dystrophy
BMI	Body mass index
CK	Creatine kinase
CTD	Connective tissue disorders
DCM	Dilatative cardiomyopathy
DMD	Duchenne muscular dystrophy
DM1	Myotonic dystrophy type 1
DOMS	Delayed-onset muscle soreness
ELISA	Enzyme-linked immunosorbent assay
FCMD	Fukuyama congenital muscular dystrophy
ICU-AW	Intensive care unit-acquired weakness
IIM	Idiopathic inflammatory myopathy
ILD	Interstitial lung disease
LGMD	Limb-girdle muscular dystrophy
MDX	Muscular dystrophy X-linked
MVC	Maximum voluntary contraction
ROM	Range of motion
T2DM	Type-2 diabetes mellitus
UTN	Urinary titin-N fragment
